# A Functional Magnetic Resonance Imaging Study of Verbal Working Memory in Young People at Increased Familial Risk of Depression

**DOI:** 10.1016/j.biopsych.2009.10.006

**Published:** 2010-03-01

**Authors:** Zola N. Mannie, Catherine J. Harmer, Philip J. Cowen, Ray Norbury

**Affiliations:** Psychopharmacology Research Unit, University Department of Psychiatry, Warneford Hospital, Oxford, United Kingdom

**Keywords:** Depression, fMRI, high-risk, n-back, working memory

## Abstract

**Background:**

Patients with depression show abnormalities in the neural circuitry supporting working memory. These abnormalities apparently persist into clinical remission, raising the possibility that they might be trait markers indicating vulnerability to depression.

**Methods:**

We studied 17 young people who had a depressed parent but no personal history of depressive illness (FH) and 15 healthy control subjects with no family history of depression. Participants performed a verbal working memory task of varying cognitive load (n-back) while undergoing functional magnetic resonance imaging scanning. We used multiple regression analyses to assess overall capacity (1-, 2-, 3-back vs. 0-back) as well as linear and quadratic modulation of cognitive demand.

**Results:**

Performance accuracy and response latency did not differ between groups, and overall capacity was similar. However, for both linear and quadratic load response activity, FH participants showed greater activation in lateral occipital cortex, superior temporal cortex, and superior parietal cortex.

**Conclusions:**

Our data suggest that, as in depressed patients, maintenance of task performance in FH participants is associated with a significant increase in the load-response activity of the cortical regions involved in working memory. This neural abnormality could form part of the predisposition to develop depressive disorders.

Major depression is associated with clinically significant deficits in cognitive function, including attention, concentration, and learning (1,2). Theoretically, these deficits could stem from abnormalities in executive function, particularly in working memory (3). Indeed, measures of verbal working memory, such as digit span (4,5) and the Paced Auditory Serial Addition Test (3,6), are reliably impaired in depressed patients; however, results from tasks of spatial working memory are less consistent (7).

The neural basis of working memory has been well characterized in imaging studies (8,9). For example, the n-back task, where subjects are asked to monitor the identity or location of a series of verbal or nonverbal stimuli and to indicate when the currently presented stimulus is the same as the one presented *n* trials previously, robustly activates areas of frontal and parietal cortex, including medial and lateral premotor cortex, cingulate cortex, dorsolateral prefrontal cortex (DLPFC) and ventrolateral prefrontal cortex, and medial and lateral posterior parietal cortex (8,9). Imaging studies of the n-back task in depressed patients have found evidence of overactivity of cortical and cingulate regions relative to control subjects during task performance and these abnormalities appear to persist in patients who have remitted with antidepressant treatment (10–12). This suggests that depressed patients allocate more processing resources to maintain task performance when using working memory.

The fact that neural overactivity during working memory persists in depressed patients in clinical remission (12) suggests that abnormalities in the neural circuitry supporting working memory might be a vulnerability marker of major depression. The aim of the present study was to explore this notion further by assessing neural responses to the n-back task in a group of young people at increased familial risk of depression but with no personal history of mood disorder. Based on the extant literature, we predicted that normal behavioral performance in at-risk subjects performing the n*-*back task would be associated with hyperactivity in cortical and cingulate regions compared with healthy control subjects.

## Methods and Materials

### Participants

We included 17 young people (14 women, 3 men), mean age 17.6 years (range 16–20 years), who had never personally suffered from major depression but who had a biological parent with a history of major depression (FH). Potential participants were assessed with the Structured Clinical Interview for DSM-IV Axis I Disorders (13) to exclude a personal current or previous history of major depression. The presence of major depression in a parent was assessed by the family history method using the participant as an informant (14). The criteria used included description of the symptoms of major depression together with the prescription of specific antidepressant treatment, either psychotherapy or medication. A history of bipolar disorder in a parent was an exclusion criterion. We also recruited 15 control subjects (11 women and 4 men), mean age 18.9 years (range 17–21 years), who were determined by the same instruments to have no current or past history of major depression and no history of depression in a biological parent or other first-degree relative (HC). All participants were right-handed, according to the Edinburgh Handedness Inventory (15), and had normal or corrected to normal vision. Participants with any personal history of Axis I and neurological disorders were excluded.

Participants were assessed on the Hospital Anxiety and Depression Scale (HADS) (16) for current emotional state and the Eysenck Personality Questionnaire-Revised (EPQ-R) (17) for the neuroticism trait. The National Adult Reading test (18) was used to estimate IQ (Table 1). All participants gave full informed consent to the study, which was approved by the local ethics committee, and received an honorarium for their participation.

### Working Memory Task Design

During functional magnetic resonance imaging (fMRI) scanning, subjects completed a letter variant of the n-back task (10). Working memory load was manipulated by using three levels of complexity: 1-, 2-, 3-back tasks. Briefly, subjects were requested to indicate whether a letter presented on the screen (the “target” stimulus) matched a previously presented letter (the “cue” stimulus). To minimize visual and phonological strategies, we used phonologically closed letters presented in upper and lower case. Thus, only the following characters were presented: b, B, d, D, g, G, p, P, t, T, v, V. Subjects were instructed to ignore the case of letters and respond by pressing a button with their right or left thumb if the target was identical or different from the cue, respectively. Subjects also performed a sensorimotor control task (0-back) during which they were required to respond to a prespecified letter (x, X). All blocks consisted of a sequence of 10 consonants varying in case. Letters were presented for 500 msec with a fixed interstimulus interval of 1500 msec. Prior to each task block, an instruction screen (0-, 1-, 2-, 3-back) was presented for 2000 msec. A 4000-msec blank screen separated the instruction from the onset of the first letter. Task blocks were separated by 8000 msec of fixation cross. Four blocks of each condition were presented in a fixed pseudorandom order (0-, 1-, 2-, 3-, 1-, 3-, 2-, 0-, 2-, 1-, 0-, 3-, 1-, 0-, 3-, 2-back). All conditions were matched for the number of target and upper/lower case letters presented. Stimuli were presented on a personal computer using E-Prime (version 1.0; Psychology Software Tools, Inc., Pittsburgh, Pennsylvania) and projected onto an opaque screen at the foot of the scanner bore, which subjects viewed using angled mirrors. Subject responses were made via a magnetic resonance imaging (MRI)-compatible keypad. Both accuracy and response latency were recorded by E-Prime. Immediately before scanning, all subjects received training with another set of stimuli to ensure they fully understood the task requirements.

### fMRI Data Acquisition

All imaging data were collected using a Siemens Sonata 1.5-T scanner (Siemens AG, Erlangen, Germany) located at the Oxford Centre for Magnetic Resonance, University of Oxford. Functional imaging consisted of 29 T2*-weighted echo-planar image (EPI) axial oblique slices that began at the cerebral vertex and encompassed the entire cerebrum and the majority of the cerebellum. Acquisition parameters were as follows: repetition time (TR)/echo time (TE) = 3000 msec/50 msec, flip angle 90°, field of view/matrix size = 192 × 192/64 × 64, slice thickness = 3 mm. These parameters were selected to optimize signal across the entire volume of acquisition. The first two EPI volumes in each session were discarded to avoid T1 equilibration effects. To facilitate later coregistration of the fMRI data into standard space, we also acquired a turbo fast low-angle shot sequence (TR = 12 msec, TE = 5.65 msec) voxel size = 1 mm^3^.

### Gray Matter Probability Maps

Each individual subject's T1-weighted high-resolution anatomic image was registered to a standard template (Montreal Neurological Institute [MNI] 152 stereotactic template) using an affine procedure with a 12-parameter fit (19). The MRI images were then segmented into three tissue classes (cerebrospinal fluid, white matter, and gray matter) (20). Gray matter probability maps were masked (i.e., nonbrain voxels set to zero) and smoothed to yield images with similar smoothness to the corresponding functional data, to minimize partial volume effects (21). Lastly, the resulting smoothed gray matter probability maps were demeaned before inclusion in the between-groups analysis.

### fMRI Data Analysis

fMRI data were preprocessed and analyzed using FSL, version 4.1 (22). Preprocessing included within-subject image realignment (23), correction for geometric EPI distortions based on an acquired B0 field map (24), nonbrain removal, and spatial normalization to a standard template (as described previously). During registration, signal loss (resulting from through-slice field gradients) was calculated and used as a cost function mask to exclude voxels where signal loss was greatest. Finally, images were spatially smoothed using a Gaussian kernel (5 mm full-width at half-maximum) and high-pass filtered (to a maximum of .008 Hz).

Analyses of data from individual subjects were computed using the general linear model with local autocorrelation correction (25). Four explanatory variables were modeled: 0-, 1-, 2-, and 3-back. These explanatory variables were modeled by convolving each trial block with a hemodynamic response function, using a variant of a gamma function (i.e., a normalization of the probability density function of the gamma function) with a standard deviation of 3 sec and a mean lag of 6 sec. In addition, temporal derivatives, estimated motion parameters (three translation and three rotation), task instruction, and anticipation period were included in the model as regressors of no interest to increase statistical sensitivity. Regression analyses modeled three mutually orthogonal characteristics of brain activation at each voxel: 1) mean overall capacity (i.e., 1-, 2-, 3-back vs. 0-back); 2) linear load response across each level of task difficulty (modeled with the following contrast: −3 − 1 1 3); and 3) a quadratic load response contrast (1 −1 −1 1) to each level of task difficulty.

Individual subject data were combined at the group level using full mixed-effects analyses (26). This mixed-effects approach enables generalization of the results beyond the sample of subjects tested. We included age, IQ, and gray matter probability maps at a given voxel as covariates (nuisance variables) to minimize the potential impact of these variables on group comparisons (21). Significant activations across the whole brain were identified using cluster-based thresholding of statistical images with a height threshold of *Z* = 2.0 and a (whole-brain corrected) spatial extent threshold of *p* < .05. In addition, to identify the main effect of task across both groups, we conducted a simple one-sample *t* test on the overall capacity, linear load response, and quadratic load response contrast images (height and extent threshold as above). These images were then used as a small volume correction to further explore significant between-group differences identified across the whole brain.

Finally, we used a region of interest (ROI) approach to investigate medial prefrontal cortex (mPFC) response as a function of task complexity. Our ROI was a 5-mm sphere centered on the coordinates (x = 0, y = 54, z = 3) described by Harvey *et al.* (10), who reported a reduced capacity to deactivate mPFC in depressed patients compared with control subjects at higher levels of n-back task difficulty.

### Behavioral and Demographic Data Analysis

Demographic and neuropsychological data were compared using Student *t* tests, Mann–Whitney *U* test, and chi-squared tests for independence where appropriate. N-back response accuracy and latency were analyzed using repeated-measures analyses of variance (ANOVAs) with group as the between-subjects factor (two levels) and complexity as the within-subjects factor (three levels). Student *t* tests were used to compare groups for the 0-back condition.

## Results

### Demographic and Neuropsychological Measures

Groups did not differ in terms of sex ratio (χ^2^ = .379, *p* = .538). We did, however, observe significant between*-*group differences in age (Mann-Whitney *U* = 62.5, *p* < .034) and predicted IQ [*t*(30) = 2.58, *p* = .015], although for both groups predicted IQ was above average (greater than one standard deviation above the mean for both groups [for details see Table 1]). Anxiety, depression, and personality ratings for one FH participant were not available. Subsequent analysis of these measures, therefore, included 16 FH and 15 HC. Family history of depression was associated with higher anxiety [*t*(29) = −3.07, *p* = .005] and depression ratings (Mann-Whitney *U* = 67.5, *p* < .037), although for both groups estimates were not clinically relevant. Groups did not differ on any personality measure as assessed by the EPQ-R (all *p*s> .05).

### N-Back Performance

Due to technical difficulties, behavioral data for two subjects (one FH) were not acquired. Subsequent analyses of these measures, therefore, included 14 HC and 16 FH. Groups were similar in terms of 0-back response accuracy [*t*(28) = 1.4, *p* = .17] and latency [*t*(28) = .497, *p* = .62]. Similarly, repeated-measures ANOVAs did not reveal significant between-group differences for either response accuracy [main effect of group; *F*(1,28) = 3.061, *p* = .09] or latency [main effect of group; *F*(1,28) = 2.18, *p* = .15]. As expected, increasing complexity was associated with reduced accuracy [main effect of complexity; *F*(2,56) = 33.36, *p* < .001] and increased response latency [main effect of complexity; *F*(2,56) = 19.70, *p* < .001]. There was no significant group × complexity interaction for response accuracy [*F*(2,56) < 1] or latency [*F*(2,56) = 2.37, *p* < .10].

### fMRI Data

#### Overall Capacity (N-Back vs. 0-Back)

**Figure 1:**
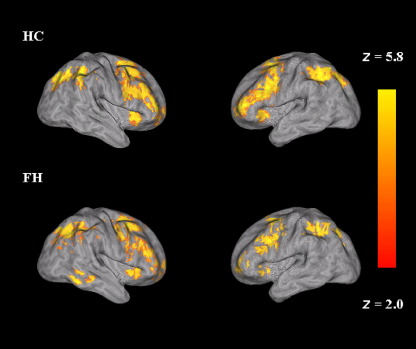
Mean overall capacity (n-back vs. 0-back) in HC (top row) and FH (bottom row). Left hemisphere is depicted on the right side of the image. Color bar reflects minimum and maximum *Z* score. FH, people who had a depressed parent but no personal history of depressive illness; HC, healthy control subjects with no family history of depression.

For each group, activations were observed in anterior cingulate, parietal, medial frontal, temporal, and occipital gyri. The regions of activation observed here are consistent with those previously reported (8,9) (Figure 1). There were no significant between-group differences in terms of overall capacity that reached statistical significance either across the whole brain or following small volume correction (i.e., masked with the main effect of overall capacity).

#### Linear Load Response Activity

**Figure 2:**
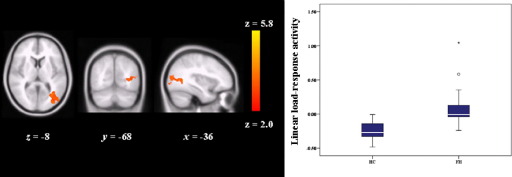
Greater linear load-response activity in FH participants as compared with HC in lateral occipital cortex. Left hemisphere is depicted on the right side of the image and the numeral indicates the location of the z dimension in the Montreal Neurological Institute space. Color bar reflects minimum and maximum *Z* score. Box plots; boxes show interquartile range; lines, the medians; limit lines, range excluding outliers. FH, people who had a depressed parent but no personal history of depressive illness; HC, healthy control subjects with no family history of depression.

**Figure 3:**
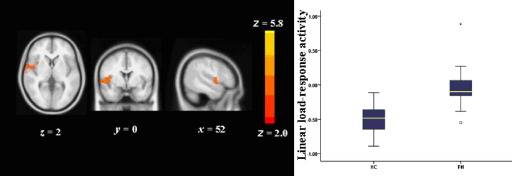
Greater linear load-response activity in FH participants as compared with HC in superior temporal gyrus. Left hemisphere is depicted on the right side of the image and the numeral indicates the location of the z dimension in the Montreal Neurological Institute space. Color bar reflects minimum and maximum *Z* score. Box plots; boxes show interquartile range; lines, the medians; limit lines, range excluding outliers. FH, people who had a depressed parent but no personal history of depressive illness; HC, healthy control subjects with no family history of depression.

For load response activity, FH participants showed significantly greater activity in lateral occipital cortex (cluster size [voxels] = 565, *p* = .009, MNI coordinates; x = −50, y = −78, z = 4) (Figure 2) and superior temporal gyrus (cluster size [voxels] = 532, *p* = .01, MNI coordinates; x = 52, y = 0, z = 0) (Figure 3). Cluster data remained significant after simultaneously controlling for depression, anxiety, and n-back performance [lateral occipital cortex, *F*(1,25) = 8.68, *p* = .007; superior temporal gyrus, *F*(1,25) = 11.95, *p* = .002]. There were no additional differences between groups using the task ROI.

Within the family history positive group, there were no significant correlations between load response activity and mood or anxiety ratings, age, IQ, or n-back performance. In the HC group, there was a significant correlation between n-back performance and blood oxygenation level-dependent (BOLD) response in lateral occipital cortex. All other associations were nonsignificant (for a complete list of Pearson's *r* and associated *p* values, see Tables S1 and S2 in Supplement 1).

#### Quadratic Load Response Activity

**Figure 4:**
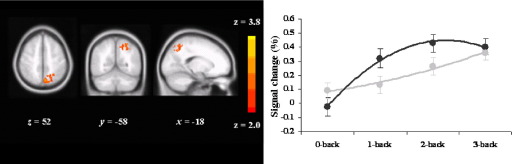
Quadratic load-response activity in FH participants as compared with HC in parietal cortex/precuneal border. Color bar reflects minimum and maximum *Z* score. Left brain is shown on the right. Numeral indicates the location of the x, y, and z dimensions in the Montreal Neurological Institute space. Plot displays mean (dark grey circles HC, light grey FH); error bars show SE; lines show best fit using a second degree polynomial. FH, people who had a depressed parent but no personal history of depressive illness; HC, healthy control subjects with no family history of depression; SE, standard error of the mean.

Family history of depression was associated with greater quadratic load response activity in superior parietal/precuneal border (cluster size [voxels] = 467, *p* = .01, MNI coordinates; x = −6, y = −68, z = 54) compared with healthy control subjects (Figure 4). Cluster data remained significant after simultaneously controlling for depression, anxiety, and n-back performance [*F*(1,25) = 19.167 *p* < .001]. There were no additional differences between groups using the task ROI.

In addition, within groups there were no significant correlations between load response activity and mood or anxiety ratings, age, IQ, or n-back performance (for a complete list of Pearson's *r* and associated *p* values, please see Tables S3 and S4 in Supplement 1).

**Figure 5:**
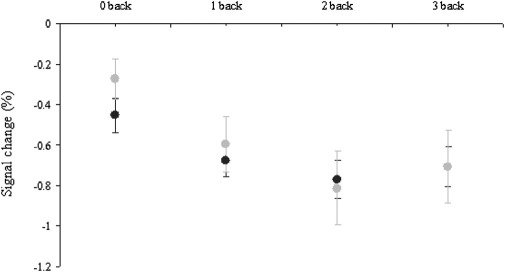
BOLD deactivation (as percent signal change) in mPFC (data extracted from a 5 mm sphere centered on x = 0, y = 54, z = 3). Plot displays mean (dark grey circles HC, light grey FH); error bars show SE. BOLD, blood oxygenation level-dependent; FH, people who had a depressed parent but no personal history of depressive illness; HC, healthy control subjects with no family history of depression; mPFC, medial prefrontal cortex; SE, standard error of the mean.

Consistent with Harvey *et al.* (10), post hoc analyses in the mPFC ROI revealed a significant main effect of task complexity [*F*(2,60) = 3.79, *p* < .028], with a pattern of decreased BOLD response as task complexity increased (Figure 5). However, there was no main effect of group [*F*(1,30) < 1], group × complexity interaction [*F*(2,60) < 1], or 0-back condition [*t*(28) = 1.375, *p* = .18].

## Discussion

Our findings indicate that young people at increased familial risk of depression exhibit overactivity of the brain networks supporting working memory, a phenomenon similar to that described for acutely and remitted depressed patients (10–12). This raises the interesting possibility that altered neural responses to a working memory task might represent a vulnerability marker of depression.

A number of limitations to the current study need to be noted. First, compared with previous studies assessing working memory in depressed patients, we included relatively young adults (mean age 18.19). It is possible, therefore, that the hyperfrontality previously reported in depressed populations (10,11), but not observed here, could be due to the younger population we studied. However, arguing against this view, the BOLD response associated with overall capacity (n-back vs. 0-back) we observed was consistent with a number of previous neuroimaging studies of working memory (see [9] for a meta-analysis), suggesting that our population of younger adults (both HC and FH) engaged the same neural networks, including prefrontal regions, as older adults. Second, our relatively small sample size (HC = 14, FH = 16) may have left us exposed to a type II error in detecting behavioral differences between the two groups (FH participants showed numerically increased response latency and reduced accuracy, although between-group differences were nonsignificant). However, between-group differences in BOLD response remained significant after controlling for age, full-scale IQ, and n-back performance. In addition, indexing subtle metrics, such as response time, in the scanning environment is challenging. Subjects are positioned supine on the scanner gantry, and stimuli are viewed via angled mirrors (or equivalent set-up) and processed in the presence of background noise. Future larger behavioral studies are required to examine working memory performance in at-risk populations and how these potential differences may be related to vulnerability to depression.

As noted above, functional imaging studies in depressed patients have found overactivity of corticolimbic circuitry during working memory tasks, generally in the presence of normal behavioral performance (10–12). As in the present study, differences between patients and control subjects became particularly apparent as cognitive load increased. For example, both Matsuo *et al.* (27) and Harvey *et al.* (10) found increased activation in depressed patients in lateral prefrontal cortex and anterior cingulate cortex during working memory tasks, while Fitzgerald *et al.* (28) and Walter *et al.* (29) also found overactivation of lateral prefrontal cortex. In addition, Rose *et al.* (7) and Harvey *et al.* (10) reported that depressed patients showed an attenuation of the reduction in anterior mPFC activity that control subjects demonstrated at the highest level of task difficulty. However, we did not observe any significant between-group differences in mPFC deactivation. Our results, therefore, suggest that resource allocation impairment (i.e., the reduced ability to deactivate counterproductive regions [mPFC] and maximize activity in regions supporting the task [DLPFC]) is not present in people at increased familial risk of depression. Future studies in recovered depressed patients are required to examine if resource allocation abnormalities persist into periods of remission.

The latter studies were conducted in acutely depressed patients, many of whom were receiving medication. However, Schöning *et al.* (12), using an n-back task in remitted, antidepressant-treated patients, also found hyperactivation of anterior cingulate cortex, though lateral prefrontal activation in these subjects did not differ from control subjects. Finally, Walsh *et al.* (11) studied 20 unmedicated depressed patients in a working memory task before and following 8 weeks fluoxetine treatment. They found that relative to control subjects, depressed patients had greater linear load response activity in inferior parietal cortex and superior temporal cortex, while a greater quadratic load response was seen in inferior frontal cortex. These differences were not attenuated by antidepressant treatment or clinical improvement, suggesting that overactivity of the neural circuitry involved in working memory performance might be a trait marker for depression.

The findings of the present study support this notion in that the increased neural activation seen in the FH participants during the n-back task cannot be attributed to antidepressant medication. The FH participants did have higher scores than control subjects on the HADS, a standard self-rating scale of anxiety and depression (16); however, the differences between the groups were small and not of clinical significance. Furthermore, covariance and correlational analyses indicated that the increased depression and anxiety ratings of the FH participants did not explain the differences in neural responses between the two groups. It should be noted, however, that our family history method of identifying at-risk individuals was not sufficiently detailed to detect parental comorbidity with, for example, anxiety disorders, which could also be implicated in the increased neural responses in the FH participants.

The brain regions in the FH participants that demonstrated neural overactivity in the present study (lateral occipital cortex, superior temporal gyrus, and superior parietal/cuneal border) do not map directly to those reported in working memory studies of depressed patients (see above). However, the between-group differences identified here do fall within brain networks known to be important for working memory performance (8,9,30). In addition, the current observation of increased superior temporal gyrus activation as a linear function of load in FH participants is very similar to that reported by Walsh *et al.* (11) in depressed patients. We would argue, therefore, that our findings support their proposal that at higher levels of cognitive demand, depressed patients show greater recruitment of an extended working memory network as a whole, presumably to maintain task performance (11).

In this respect, it is clearly of interest that people at risk of depression through increased familial risk appear to show a similar phenomenon in the neural response to working memory as acute and remitted depressed patients. In another group of FH participants, we have previously found impaired activation of anterior cingulate cortex in response to an emotional Stroop task, suggesting that young people at risk of depression have impairment in the neural circuitry involved in integrating emotional and cognitive information (31). The present findings suggest that abnormalities in the neural circuitry underpinning working memory and executive function might also form part of the familial risk of experiencing depression. Further work will be needed to test this hypothesis and to see if changes in the neural basis of working memory might specifically predict those most likely to experience clinical illness and whether they might perhaps also form targets for treatment.

## Figures and Tables

**Table 1:** Participant Characteristics and N-Back Performance

	FH (*n* = 17)	HC (*n* = 15)
Age^a^	17.5 (1.2)	19.1 (1.3)
Gender: F/M	14/3	11/4
IQ^a^	116.2 (4.0)	119.0 (3.0)
HADS-D^a^^,^^b^	2.5 (2.3)	1.1 (.9)
HADS-A^a^^,^^b^	8.1 (4.3)	4.7 (2.7)
EPQ-N^b^	12.4 (5.3)	9.6 (4.4)
0-Back^c^		
Accuracy (%)	84.8 (16.4)	86.1 (20.6)
Response latency (msec)	465.6 (85.5)	474.4 (64.1)
1-Back^c^		
Accuracy (%)	82.6 (15.4)	87.2 (12.3)
Response latency (msec)	485.1 (71.8)	512.4 (89.6)
2-Back^c^		
Accuracy (%)	79.0 (19.2)	87.3 (23.5)
Response latency (msec)	601.8 (122.1)	630.0 (139.3)
3-Back^a^^,^^c^		
Accuracy (%)	57.2 (15.4)	62.1 (13.8)
Response latency (msec)	552.3 (74.7)	664.5 (177.7)

Given values are mean with standard deviation in parentheses. EPQ-N, Eysenck Personality Questionnaire-Revised; F, female; FH, people who had a depressed parent but no personal history of depressive illness; HADS-A, Hospital Anxiety and Depression Scale, anxiety subscale; HADS-D, Hospital Anxiety and Depression Scale, depression subscale; HC, healthy control subjects with no family history of depression; IQ, intelligence quotient; M, male.

a *p* < .05.

b HADS and EPQ-N include 16 FH and 15 HC.

c N-back performance measures include 16 FH and 14 HC.
