# Supplementary material for: A Functional Magnetic Resonance Imaging Study of Verbal Working Memory in Young People at Increased Familial Risk of Depression
Source: Biol Psychiatry. 2010 Mar 1;67(5):471–7. doi: 10.1016/j.biopsych.2009.10.006 (PMC2890050; doi:10.1016/j.biopsych.2009.10.006)
Supplement: Supplement 1 [file mmc1.pdf]

Supplemental Information

Table S1. Linear load response activity. Displayed are Pearson's *r* values and associated significance in family history positive participants. Lateral occipital cortex and superior temporal gyrus refer to the significant clusters identified in the linear load response activity contrast.

	N-back performance	Full scale IQ		Lateral occipital cortex		Superior temporal gyrus		Age	
		r	*P*	*r*	*P*	*r*	*P*	*r*	*P*
N-back performance	1	0.123	0.649	-0.284	0.286	-0.276	0.3	0.058	0.83
Full scale IQ		1		-0.028	0.91	-0.149	0.569	0.16	0.54
Lateral occipital cortex				1		*N/A*	*N/A*	0.137	0.6
Superior temporal gyrus						1		0.195	0.454
Age								1	

N/A, not applicable.

Table S2. Linear load response activity. Displayed are Pearson's *r* values and associated significance in healthy controls. Details as in Table S1.

	N-back performance	Full scale IQ		Lateral occipital cortex		Superior temporal gyrus		Age	
		r	*P*	*r*	*P*	*r*	*P*	*r*	*P*
N-back performance	1	0.249	0.39	-0.605	*0.02	-0.477	0.085	0.162	0.518
Full scale IQ		1		-0.027	0.95	-0.295	0.285	0.041	0.885
Lateral occipital cortex				1		*N/A*	*N/A*	-0.202	0.471
Superior temporal gyrus						1		-0.019	0.945
Age								1	

* $P < 0.05$.

Table S3. Quadratic load response activity. Displayed are Pearson's *r* values and associated significance in family history positive participants. Parietal/precuneal border refers to the significant cluster identified in the quadratic load response activity contrast.

	N-back performance	Full scale IQ		Parietal/precuneal border		Age	
		r	*P*	*r*	*P*	*r*	*P*
N-back performance	1	0.123	0.649	0.126	0.643	0.058	0.83
Full scale IQ		1		0.026	0.921	0.16	0.83
Parietal/precuneal border				1		-0.192	0.46
Age						1	

Table S4. Quadratic load response activity. Displayed are Pearson's *r* values and associated significance in healthy controls. Details as in Table S3.

	N-back performance	Full scale IQ		Parietal/precuneal border		Age	
		r	*P*	*r*	*P*	*r*	*P*
N-back performance	1	0.249	0.39	-0.196	0.502	0.162	0.518
Full scale IQ		1		0.059	0.835	0.041	0.885
Parietal/precuneal border				1		-0.188	0.502
Age						1	
